# Development and validation of a deep learning algorithm for pattern-based classification system of cervical cancer from pathological sections

**DOI:** 10.1016/j.heliyon.2023.e19229

**Published:** 2023-08-21

**Authors:** Wei Tian, Siyuan Sun, Bin Wu, Chunli Yu, Fengyun Cui, Huafeng Cheng, Jingjing You, Mingjiang Li

**Affiliations:** a Department of Gynecology, Shandong Provincial Hospital Affiliated to Shandong First Medical University, Jinan, Shandong, China; b Gynecology Laboratory, Shandong Provincial Hospital, Jinan, Shandong Province, China; c AI Research Group, Yi hui Ebond (Shandong) Medical Technology Company Limited, Jinan, Shandong, China; d Department of Gynecology, Taian City Central Hospital, Taian, Shandong, China; e Department of Pathology, Shandong Provincial Hospital Affiliated to Shandong First Medical University, Jinan, Shandong, China

**Keywords:** Pattern-based classification system according to silva, Endocervical adenocarcinomas, Whole slide images, ResNet50, Deep learning system

## Abstract

**Background:**

Multi-center research has demonstrated that adopting Silva's pattern-based classification system (SPBC) enhances the clinical prognosis and facilitates hierarchical management of patients with endocervical adenocarcinomas (EAC). However, inconsistencies in SPBC can arise due to variations in pathologists' experience levels. Thus, the implementation of standardized decision-making tools becomes crucial to enhance the practicality of SPBC in clinical diagnosis and treatment.

**Methods:**

We enrolled a total of 90 patients with EAC in this study, of which 63 were assigned to the training group, and the remaining 27 were allocated to the validation group. To create and validate the prediction models for SPBC, we utilized a deep learning system (DLS) and calculated the area under the receiver operating characteristic curve (AUC).

**Results:**

In Silva pattern classification, ResNet50 achieved an average accuracy of 74.36% (63.64% for pattern A, 55.56% for pattern B, and 89.47% for pattern C respectively). Moreover, in test set, ResNet50 achieved an AUC of 0.69 for pattern A, 0.58 for pattern B, and 0.91 for pattern C.

**Conclusions:**

We successfully established a DLS for SPBC, which holds the potential to aid pathologists in accurately classifying patients with EAC.

## 1 Introduction

Cervical cancer ranks as the fourth most prevalent malignant tumor in females worldwide, with endocervical adenocarcinoma (EAC) comprising 10%–25% of cases. The National Comprehensive Cancer Network Clinical Practice Guidelines (NCCN) in Oncology recommend treatment strategies for SCC and EAC primarily based on the Federation International of Gynecology and Obstetrics (FIGO) staging, which relies on depth of invasion and tumor size [1,2]. However, accurately evaluating EAC poses challenges due to the difficulty in measuring crucial pathological parameters in FIGO and the limited consistency in EAC specimens. In 2015, Roma et al. proposed a cervical adenocarcinoma pattern classification system (The pattern-based classification system according to Silva, SPBC), namely Silva pattern classification, based on the theory of EAC risk stratification according to the mode of tumor invasion proposed by Silva and his colleagues [3]. SPBC is based on the invasion pattern of the tumor and proposed as an alternative classification system to evaluate EAC, which doesn't require measuring the depth of invasion or distinguishing between in situ adenocarcinoma and invasive adenocarcinoma with lighter lesions. Despite the promising application prospects of SPBC and its potential to complement FIGO staging and NCCN guidelines, further revision is still necessary.

At present, the research object of SPBC is limited to common cervical adenocarcinoma, and other special histological subtypes of invasive cervical adenocarcinoma have not been included in the study, which limits the application of SPBC [4]. As analysis of SPBC has only been reported with limited cohorts, both the reproducibility and the prognostic value of this methodology need to be confirmed and validated through clinical practice [5]. Therefore, effective clinical verification and reasonable expansion of SPBC is an unavoidable opportunity and challenge for pathologists and clinicians.

In clinical practice, accurately classifying SPBC of EAC is crucial for pathologists. However, the current qualitative visual analysis of microscopic images is a time-consuming and tedious process, lacking objective standards [6,7]. The diagnostic results for the same case can vary among different pathologists, and with the rising incidence of cancer, it becomes increasingly time-consuming and overwhelming to handle the diagnosis and grading of cancer cases. To draw a complete diagnosis, pathologists must go through a large number of glass slides, often including conventional H&E slides and additional immunohistochemical stains [8]. As a result, exploring an expert level automatic system for pathological specimen evaluation seems more and more necessary.

As a subfield of machine learning, deep learning lies in the establishment of a neural network that simulates the human brain for analytical learning, which mimics the mechanisms of the human brain to interpret data, such as images, sounds and texts [9]. At present, convolution neural network (CNN) is the most widely used deep learning network, which uses a mathematical operation called convolution to iteratively adjust the weight of training samples to extract relevant features from training samples [10]. During the construction of the pathological diagnosis model, combining the CNN algorithm with a meta-heuristic algorithm can optimize hyperparameters and enhance the performance of the proposed deep learning system.

In our study, our main objective is to develop an EAC pathological classification model based on SPBC using a DLS that combines ResNet50 as a patch-level classifier and OpenCV as a WSI-level classifier. This model is anticipated to be a valuable resource in assisting clinical pathologists to enhance the pathological diagnosis of EAC.

## 2 Methods

### 2.1 Data acquisition

Our study included 90 patients with EAC who received treatment at the hospital between June 2011 and August 2021. All patients had to meet the following criteria for inclusion: (1) histopathological diagnosis of primary cervical invasive adenocarcinoma, (2) availability of a tumor specimen obtained through total hysterectomy, cervical resection, or cervical conization, and (3) comprehensive medical records and pathological data. The exclusion criteria comprised: (1) presence of other primary malignant tumors, and (2) prior receipt of adjuvant therapy before surgery. The study was approved by the Ethical Committee of the Provincial Hospital of Shandong First Medical University (protocol number: SWYX 2022-127).

EAC was divided into three modes: pattern A tumor with well-differentiated structure, expansive growth, non-destructive interstitial, and lymphatic vascular space infiltration (LVSI); pattern B tumor with focal interstitial destructive infiltration based on pattern A, which may be accompanied by LVSI; and pattern C tumors showed diffuse destructive interstitial infiltration, accompanied by obvious interstitial fibrous hyperplasia, sclerosing reactions, and inflammatory reactions, which are common in LVSI [9].

## 3 Pathologic examination

**Fig. 1:**
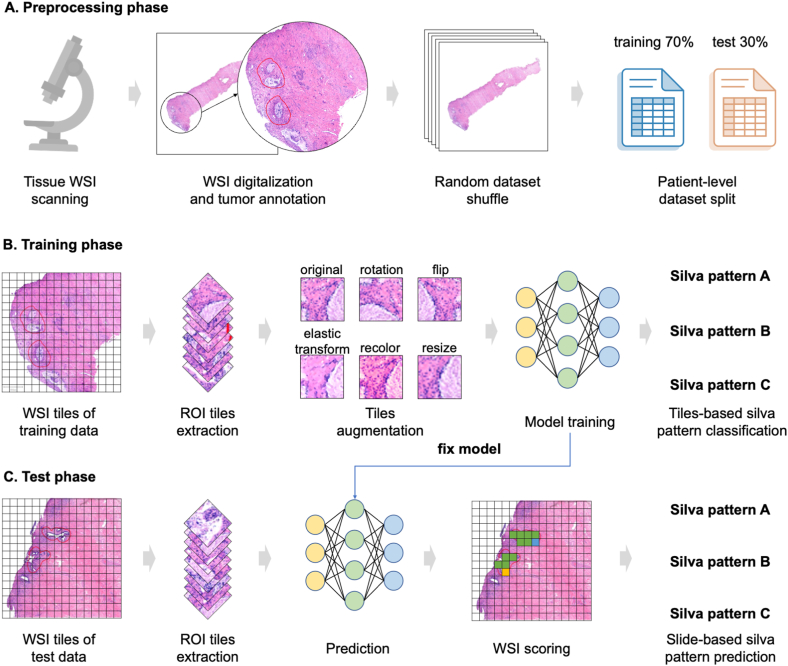
The framework of the deep learning approach. A. Preprocessing phase; B. Training phase; C. Test phase.

All surgical specimens included in this study were sliced and stained with hematoxylin-eosin for pathological evaluation. Two experienced pathologists reviewed the sections of the pathological specimens following the pattern-based classification system. They described the microscopic characteristics of the tumor tissue in each case and assigned them to one of the three levels: A, B, or C. In cases of disagreements, a third pathologist evaluated the sections, and a consensus was achieved through joint discussion. Whole slide images (WSIs) provide a more objective and quantitative review of sections and are increasingly used in histopathological diagnosis [11,12]. The WSIs of the EAC specimens were digitalized using the Provincial Hospital of Shandong First Medical University Central Lab platform. Quality control of WSIs was performed manually, and slides that were digitalized abnormally were excluded from this study. Each WSI was magnified 40 times and stored in tagged image file format (tiff format). The research procedure is illustrated in Fig. 1, providing an overview of the study framework.

## 4 Regions of interest delineation

In our study, we identified regions of interest (ROIs) as primary tumor areas in whole slide images (WSIs). These ROIs were initially delineated by a subspecialist and then thoroughly reviewed by expert pathologists using QuPath software (https://qupath.github.io/). Using the annotation panel of QuPath, users were able to outline ROIs by painting polygon shapes, which were then superimposed on the original WSI and saved as GeoJSON format files containing the coordinate information defining the polygon boundaries. Despite using the same staining protocol for all slides, differences in contrast and brightness between WSIs could still lead to heterogeneity. For instance, WSIs with the same Silva pattern might appear brighter or darker at times. This heterogeneity might negatively impact subsequent deep learning analyses and lead to model overfitting. To mitigate this issue, we applied histogram normalization to all WSIs, using a histogram matching algorithm in Python to achieve histogram consistency before further processing. The WSI template was selected by a pathologist from patients with EAC, who were stained and digitized under optimal conditions. Following preprocessing, WSIs were divided into the training and test sets at a 7:3 ratio, maintaining patient-level separation (Fig. 1A). This ensured that WSIs from a single patient were only present in either the training or test set to prevent any information leakage.

### 4.1 Tiles extraction and data augmentation

The WSIs utilized in this study were extremely large, often in the gigabyte range, posing computational challenges for analysis. To ensure efficient processing, we employed the OpenCV library in Python to crop the massive WSIs into smaller, more manageable tiles sized at 512 x 512 pixels, thereby reducing the spatial dimensions for input into the deep learning model. Since the tumor regions annotated by pathologists had irregular boundaries that did not align with tile edges, we needed to assign labels to each tile based on the percentage of tumor area coverage. Only tiles containing at least 80% tumor area were designated as positive samples, inheriting the annotation labels from the original WSIs. Tiles with less than 80% tumor area were excluded to avoid introducing noise and ambiguity in the training data (Fig. 1B).

To counter overfitting and enhance the model's ability to generalize, we employed data augmentation techniques on the tumor-containing tiles during the training phase. Various augmentations were applied to the training tiles, including random rotations of 90, 180, and 270° to introduce orientation variations, horizontal/vertical flips to mirror images, elastic transformations to alter shape, color variations to shift hues, and resizing to adjust dimensions (Fig. 1B). This comprehensive data augmentation strategy was designed to artificially increase diversity within the limited tumor tile training set. The prevention of overfitting was of utmost importance to ensure accurate generalization to new patients.

### 4.2 ResNet50 as tile-level classifier

**Fig. 2:**
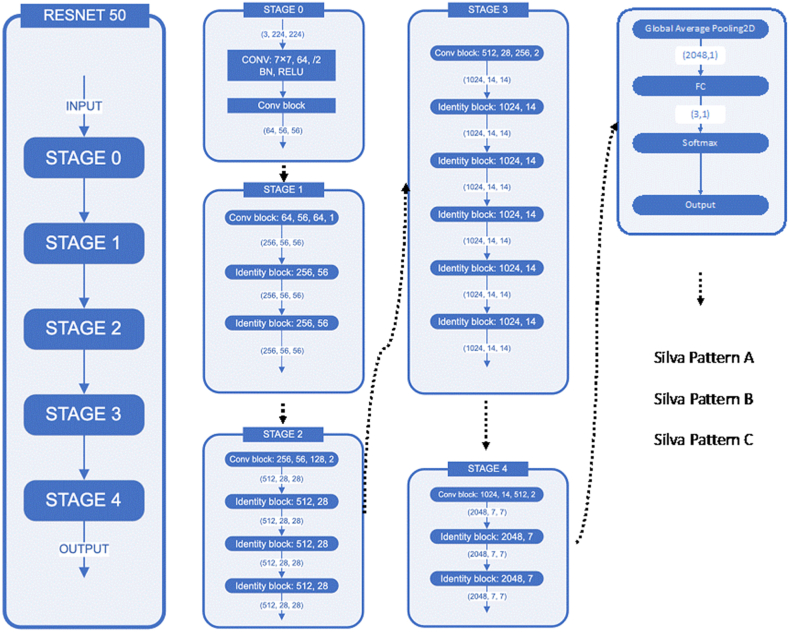
The process of model training. ResNet50 was trained on an Nvidia GTX 1060 GPU using the batch strategy, and it took over 24 h to complete the training procedure. Throughout the training step, the tile-level strategy was employed, which involved training ResNet50 to evaluate each individual tile with its corresponding ground-truth information from the WSI. To ensure validation during the training process, a portion of the training set was set aside for tiles classification validation, with multi-class cross-entropy serving as the loss function.

For classifying tiles into A, B, or C Silva patterns, we selected a pre-trained ResNet50 model and fine-tuned it using transfer learning. As shown in Fig. 2, ResNet50 consists of a feature extraction portion with five stages of convolutional and normalization layers that identify high-dimensional features representing each Silva pattern from the input tiles. Following these convolutional stages, a global average pooling 2D layer is employed to spatially condense the features by calculating the average value over each feature map. The resulting output is then fed into a fully connected layer, which interprets the features and projects them into a lower-dimensional space. Finally, a SoftMax activated fully connected layer converts these features into probability scores for the three Silva pattern classes.

During the training process, the model was fed with tiles extracted from the WSIs in the training set to predict their corresponding Silva labels (Fig. 1C). To optimize the cross-entropy loss, successive training iterations were conducted on an Nvidia GTX 1060 GPU using a batch approach, which took over 24 h to fully converge. Fine-tuning the pre-trained ResNet50 with tiles enabled the transfer of robust visual features from natural images, effectively adapting the classifier for accurate Silva pattern recognition. As a result, the optimized model demonstrated the capability to effectively classify new WSIs based on the extracted tile-level features.

During testing, WSI were classified by averaging the predicted probabilities across tiles extracted from each slide. For each 512x512 pixel tile fed into the fine-tuned ResNet50 model, the output was a 3-element vector [p_a_, p_b_, p_c_], representing predicted probability scores for Silva patterns A, B, and C respectively. By aggregating the tile-level probability scores, overall, Silva pattern predictions were obtained for entire WSIs:

Equation 1

$${Score}_{wsi}=\left(\sum_{i}p_{ia}+p_{ib}+p_{ic}\right)/n$$

This approach allowed us to harness the complete context of the WSI by combining predictions from multiple glimpse views provided by the sampled tiles. The ResNet50 model, optimized on tile-level features, could then be effectively used to categorize new patient WSIs into the most likely Silva prognostic class with robustness and accuracy.

### 4.3 Receiver operating characteristic curves

Receiver Operating Characteristic (ROC) curves are graphical representations of the response to the same signal stimulus, obtained under several different criteria. The horizontal axis denotes the false positive rate, while the vertical axis represents the true positive rate. The method's accuracy is determined by its proximity to the upper left corner. To compare the diagnostic value, we calculated the Area Under the ROC Curve (AUC) for each test. A higher AUC indicates a greater diagnostic value for the test.

### 4.4 Model evaluation and statistical analysis

Since the Silva pattern classification involved multiple classes, we calculated and visualized the confusion matrix to evaluate the model's performance. To further assess the model, we conducted ROC curve analysis and calculated the area under the curve (AUC). Since this was not a binary classification task, we analyzed each Silva pattern separately. To determine the approximate regions for the deep learning model, we utilized the QuPath software. Additionally, we employed the OpenCV library in Python to crop the massive WSIs into smaller, more manageable tiles, with dimensions of 512 x 512 pixels, thereby reducing the spatial dimensions for input into the deep learning model. Pathologists can use the results of DLS as a reference to provide a typing diagnosis and improve the diagnostic accuracy through https://github.com/MingjiangLi2024/SilvaPatternDeepLearning.

Statistical analyses using SPSS (version 26.0), QuPath (version 0.4.3), and Python (version 3.5). ResNet50 was fine-tuned on an Nvidia GTX 1060 GPU using transfer learning for Silva pattern classification during model training. The predictive performance of the model was evaluated using the AUC value obtained from the ROC curve. In addition, we used the AUC value calculated by the ROC curve to assess the model's predictive performance. The p-values in the multivariable analyses were computed using the Chi-square test, and a p-value of less than 0.05 was deemed significant.

## 5 Results

### 5.1 Analysis of patient characteristics

The detailed patient characteristics are shown in Table 1. According to the diagnostic criteria of SPBC, 90 patients with EAC, 22 (24.5%) patients were diagnosed with Silva pattern A, 12 (13.3%) patients with Silva pattern B, and 56 (62.2%) with Silva pattern C. Seventy-seven (85.6%) patients were diagnosed at FIGO stage I, 13 patients at stage FIGO Ⅱ. 31(34.4%) patients had lymph vascular space invasion, and only three (3.3%) patients had positive lymph nodes. The differences in LVSI among the three patterns were statistically significant (*p* < 0.05). Most patients underwent laparotomy (n = 82, 91.1%), and 48 (53.3%) patients received adjuvant therapy after surgery.

**Table 1:** Characteristics of patients.

Characteristic	Total (n = 90)	Development group (n = 63)	Validation group (n = 27)	p value
Age, years				0.833
≤40	22	15(23.8)	7(25.9)	
>40	68	48(76.2)	20(74.1)	
FIGO stage (2018)				0.794
I	77	53(84.1)	24(88.9)	
II	13	10(15.9)	3(11.1)	
Silva pattern				0.245
A	22	15(23.8)	7(25.9)	
B	12	6(9.5)	6(22.2)	
C	56	42(66.7)	14(51.9)	
Histological grade				0.742
I (well differentiated)	16	12(19.0)	4(14.8)	
II (moderately differentiated)	38	25(39.7)	13(48.1)	
III (poorly differentiated)	36	26	10	
Status of lymph node				0.551
Positive	3	3(4.8)	0(0.0)	
Negative	87	60(95.2)	27(100%)	
Status of resection margins				0.551
Positive	3	3(4.8)	0(0.0)	
Negative	87	60(95.2)	27(100%)	
Parametrium involvement				0.317
Yes	5	5(7.9)	0(0.0)	
No	85	58(92.1)	27(100)	
LVSI				＜0.001
No	59	57(90.5)	2(7.4)	
Yes	31	6(9.5)	25(92.6)	
Stromal invasion				0.152
Superficial 1/3	35	22(34.9)	13(48.1)	
Middle 1/3	29	19(30.2)	10(37.0)	
Deep 1/3	26	22(34.9)	4(14.8)	
Tumor size, cm				0.867
<2	42	28(44.4)	14(51.9)	
2 ≤size <4	26	19(30.2)	7(25.9)	
4 ≤size <5	17	12(19.0)	5(18.5)	
≥5	5	4(6.3)	1(3.7)	
Operation method				0.701
Laparotomy	82	58(92.1)	24(88.9)	
Laparoscopy	5	3(4.8)	2(7.4)	
Transvaginal	3	2(3.2)	1(3.7)	
Adjuvant therapy				0.600
None	42	29(46.0)	13(48.1)	
Chemotherapy	41	28(44.4)	13(48.1)	
Radiotherapy				
Chemotherapy and radiotherapy	7	6(9.5)	1(3.7)	

Values are presented as n (%). FIGO, International Federation of Gynecology and Obstetrics; LVSI, lympho-vascular space invasion.

The training set comprised, 63(70%) patients and, 84(68.3%) slides. One-hundred and twenty-three WSIs were included in our study, and tile extraction resulted in a total of 77,150 tiles. Detailed information on the training and test sets is provided in Table 2.

**Table 2:** Training and test set.

Silva pattern	Training Set				Test Set			
	Pattern A	Pattern B	Pattern C	Total	Pattern A	Pattern B	Pattern C	Total
Patient level	15	6	42	63	7	6	14	27
Slide level	16	12	56	84	11	9	19	39
Tile level	1525	843	50571	52939	413	3160	20638	24211

### 5.2 Model training and validation based on DLS

**Fig. 3:**
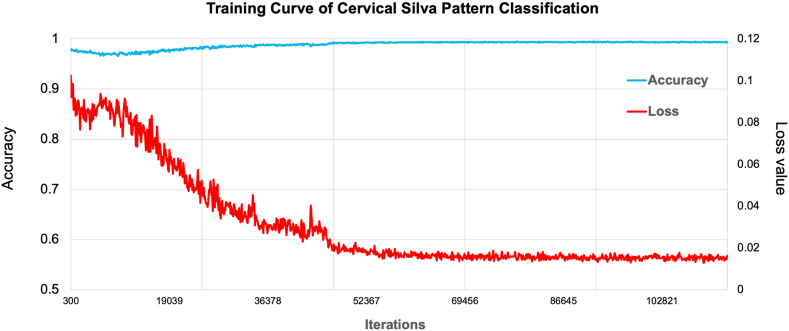
The training curves. Each point on the X-axis represents a small iteration, with the left side of the Y-axis denoting accuracy and the right side representing the loss value.

The DLS was trained on all specimens after auto-marking. To enhance the system's capability in distinguishing tumors from benign tissues, additional patches were sampled from hard-negative areas. Tiles from the training set were then fed into the model for Silva pattern prediction until model convergence, utilizing Python and employing multi-class cross-entropy as the loss function. During the test phase, the WSI was scored based on the average scores calculated from the tiles generated from the slide. As depicted in Fig. 3, the training curve represents a cyclic iterative process. Each point on the X-axis represents a small iteration, with the left side of the Y-axis denoting accuracy, and the right side indicating the loss value. When the final convergence is achieved, the iteration concludes, indicating that we have acquired the information.

### 5.3 Evaluation Prediction Performance of DLS

**Fig. 4:**
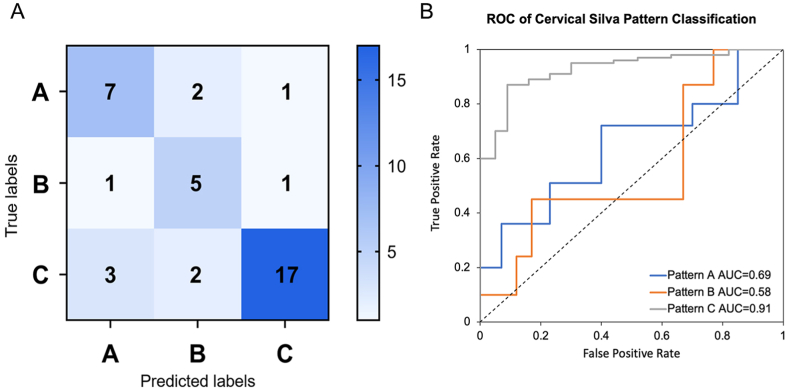
Evaluation Prediction Performance of DLS. A. Confusion matrix in test set, Average accuracy= (7 + 5+17)/39 = 74.36%, accuracy (pattern A) = 7/11 = 63.64%, accuracy (pattern B) = 5/9 = 55.56%, accuracy (pattern C) = 17/19 = 89.47%. B. ROC curve in test set, ResNet50 achieved an AUC of 0.69 for pattern A, 0.58 for pattern B, and 0.91 for pattern C.

Given the high incidence of EAC, it has great clinical value in reducing the workload for pathologists. In the test setting, our DLS achieved an AUC of 74.36% (63.64% for pattern A, 55.56% for pattern B, and 89.47% for pattern C respectively). The detailed model performance is shown in the confusion matrix in Fig. 4A.

The ROC curves of each Silva pattern are listed in Fig. 4B, and ResNet50 achieved an AUC of 0.69 for pattern A, 0.58 for pattern B, and 0.91 for pattern C. From the result, we could see the highest diagnostic accuracy is pattern C. Patterns A and B also have predictive values if the threshold is properly set.

## 6 Discussion

We have successfully developed an automated classification method for cervical adenocarcinoma, which demonstrates comparable performance to pathologists. To the best of our knowledge, this marks the first application of a deep learning algorithm to Silva's pattern-based classification system. Our approach involves training the algorithm with semi-supervised data, making it a valuable decision support tool that alleviates pathologists' workload and provides treatment guidance. Therefore, the DLS can serve as an effective pre-screening tool in the pathology department, streamlining the diagnostic process and enhancing overall efficiency.

In contrast to FIGO staging, the pattern-based classification system focuses on the range of destructive stromal glands to distinguish between the risk of LNM and recurrence in invasive EAC. Since the pattern-based classification system was proposed in 2013 [13], researchers have continuously reported on cases to verify its ability to predict LNM and recurrence [[13], [14], [15], [16], [17], [18], [19]]. In 2020, the NCCN initially mentions this 3-tier system in its footnote, indicating that the system has clinical applications [1]. It also has different degrees of adaptability to other types of cervical cancer, including malignant epithelial tumors in other organs. Current research shows that the classification system resolves some problems encountered in FIGO staging of adenocarcinoma and proposes treatment recommendations tailored to EAC. It exempts pelvic lymphadenectomy for low-risk LNM and recurrence patients and reduces the possibility of surgical complications, thereby improving the quality of life of patients. Therefore, the exploration of the genomic difference between the 3-tier patterns of EAC tumors is of clinical importance. The genomic discrepancy between non-destructive and destructive invasive tumors is definite; this difference further validates the effectiveness of the pattern-based classification system. Currently, pattern-based classification systems are only applied to the usual type, which is far beyond practical clinical expectations. Although several studies have explored other application types, a consensus remains in air [20]. Considering that pattern-based classification systems have thus far been examined using retrospective studies, prospective studies are needed to further verify their clinical applicability.

In the pathology department of cervical cancer, the traditional diagnosis method is to directly observe the pathological tissue sections under a high-power microscope, then manually adjust the magnification and range of the visual field to observe the tissue area, find the suspected focus area, observe the morphology and characteristics of the nucleus, and then obtain the diagnostic results. The whole process, from whole to part, from low-power to high-power, from tissue structure to cell morphology, is extremely time-consuming and labor-intensive, and can easily cause fatigue and is highly repetitive for doctors. Owing to limitations of the human visual system, the low-dimensional features of the pathological image could not be observed by the naked eye, resulting in a lack of consistency and objectivity of diagnosis.

With the development of automatic pathological scanning technology and artificial intelligence technology, the image location method is used to locate the suspected focus area automatically and quickly on the entire section obtained by scanning, which can save reading time and improve work efficiency. Many deep learning-based algorithms have emerged in the field of pathology images to help pathologists quickly and effectively observe and review digital full slices to detect and locate areas of suspected lesions. DLS has already emerged as a useful tool in many areas of medicine [21], such as radiology [22], ophthalmology [23,24], dermatology [25] and pathology [26]. Deep learning can obtain deeper image features and maintain the global spatial information of the image. Currently, some algorithms directly classify and diagnose super-enriched slice images, such as Google's LYNA and Baidu's NCRF methods for breast cancer classification and diagnosis [27]. U-Net is based on Res Net to automatically locate and segment the suspected lesion area in full cervical cancer slices. All existing methods only consider part of the features of the image when identifying and grading pathological diseases, ignoring the multi-level and multi-scale features of the image, resulting in low classification accuracy and poor generalization ability. However, for the subjectivity and difficulty of the Silva model classification, there were inconsistencies even in the expert group. These inconsistencies highlight the need to improve the diagnostic accuracy of cervical cancer typing further. One possible solution is to develop a system that can predict clinical risks more accurately than manual typing. This deep learning model can identify lesions that are not obvious or cannot be detected by the human eye and may help improve the accuracy of diagnosis.

Over the years, several key studies have explored survival predictions based on DL for several cancer types. To date, the FDA has approved more than a dozen DL methods for clinical use in radiology such as DL-based analysis of CT data, which was performed in a 2019 lung cancer screening trial [28]. Bychkov et al. showed that using only H&E-stained tissue microarrays could predict the 5-year disease-specific survival of CRC patients [29]. Courtiol et al. predicted OS in a large cohort of patients with malignant mesothelioma and visualized the histological features associated with long or short survival identified by the DL network [30]. This example shows that the DL network can be applied directly from histology to clinical diagnosis and treatment. Additionally, this method may contribute to the development of new diagnoses and treatments in the future.

Currently, few studies have discussed the clinical significance of DL-based clinical prediction based on biopsy. Although large prospective trials have used other prognostic biomarkers to evaluate clinical endpoints such as the use of Oncotype DX in breast cancer trials [26], there are still no clinical endpoint studies that incorporate DL survival prediction into the clinical workflow. Most scholars believe that deep learning is essentially more like a “black box”. Although the algorithm and model-building process can be explained, the decision-making process of the model is not transparent to pathologists. Technological advances are expected to improve the development and performance of DLS in the field of digital pathology in the future.

### 6.1 Limitations

Our study has several limitations that warrant acknowledgment. Firstly, we only used one slice sample from each clinical case, potentially limiting its representation of the entire tissue. Secondly, the correlation between SPBC and clinical outcomes was not assessed, which could offer valuable prognostic insights from our classification system. Thirdly, the research was confined to a single hospital, potentially affecting the generalizability of our findings. External validation from multiple centers is crucial to bolster the model's robustness. Furthermore, the impact of Rescan samples on the model's performance requires thorough evaluation in future work. Additionally, reporting the number of slides and samples used in the analysis, along with a comprehensive range of statistical indicators, adheres to standard practices in the field. Ultimately, evaluating the clinical utility of our approach in real-world settings is essential to ascertain its practical applicability and potential benefits.

## 7 Conclusions

In conclusion, we have introduced a DLS designed for Silva's pattern-based classification system, which used a DLS that combines ResNet50 as a patch-level classifier and OpenCV as a WSI-level classifier, significantly alleviating the burden on pathologists. This DLS has the potential to revolutionize the clinical workflow of cervical cancer in the near future. However, to validate these assumptions, large-scale multi-center trials are necessary. Moving forward, our future work will focus on conducting extensive multi-center trials to assess the diagnostic and clinical impact of the DLS. The aim is to enhance the accuracy and consistency of SPBC, ultimately improving the clinical diagnosis and treatment for patients.

## Declarations

### Author contribution statement

Wei Tian: Conceived and designed the experiments; Performed the experiments; Wrote the paper.

Siyuan Sun; Bin Wu; Huafeng Cheng: Analyzed and interpreted the data.

Chunli Yu; Fengyun Cui; Jingjing You: Contributed reagents, materials, analysis tools or data.

Mingjiang Li: Conceived and designed the experiments; Wrote the paper.

### 7.1 Data availability statement

Data associated with this study has been deposited at Data/References included in article. DLS as a reference could be obtained through https://github.com/MingjiangLi2024/SilvaPatternDeepLearning.

### Additional information

No additional information is available for this paper.

## Funding information

No funding was received.

## Declaration of competing interest

The authors declare that they have no known competing financial interests or personal relationships that could have appeared to influence the work reported in this paper.
